# Hydrothermal Synthesis of TiO_2_ Aggregates and Their Application as Negative Electrodes for Lithium-Ion Batteries: The Conflicting Effects of Specific Surface and Pore Size

**DOI:** 10.3390/ma14040916

**Published:** 2021-02-15

**Authors:** Saida Mehraz, Wenpo Luo, Jolanta Swiatowska, Boudjema Bezzazi, Abdelhafed Taleb

**Affiliations:** 1 Unité de Recherche Matériaux, Procédés et Environnement, M’hamed Bougara de Boumerdès University, Bourdès 35000, Algeria; s.mehraz@univ-boumerdes.dz (S.M.); bbezzazi@urmpe.dz (B.B.); 2 Chimie ParisTech—CNRS, Institut de Recherche de Chimie Paris, PSL Research University, 75005 Paris, France; wenpo.luo@chimieparistech.psl.eu (W.L.); jolanta.swiatowska@chimieparistech.psl.eu (J.S.); 3 Sorbonne Université, 4 Place Jussieu, 75231 Paris, France

**Keywords:** lithium-ion batteries, TiO_2_ aggregates, nanoparticles assembly

## Abstract

TiO_2_ aggregates of controlled size have been successfully prepared by hydrothermal synthesis using TiO_2_ nanoparticles of different sizes as a building unit. In this work, different techniques were used to characterize the as-prepared TiO_2_ aggregates, e.g., X-ray diffraction (XRD), X-ray photoelectron spectroscopy (XPS), Brunauer, Emmett and Teller technique (BET), field emission gun scanning electron microscopy (FEGSEM), electrochemical measurements etc. The size of prepared TiO_2_ aggregates varied from 10–100 nm, and their pore size from around 5–12 nm; this size has been shown to depend on synthesis temperature. The mechanism of the aggregate formations was discussed in terms of efficiency of collision and coalescence processes. These newly synthetized TiO_2_ aggregates have been investigated as potential negative insertion electrode materials for lithium-ion batteries. The influence of specific surface areas and pore sizes on the improved capacity was discussed—and conflicting effects pointed out.

## 1. Introduction

In materials science, recent research has been mainly devoted to the development of innovative strategies to prepare nanomaterials with desired properties. Among these strategies, nanoparticle agglomeration appears to be a promising way to obtain materials with controlled architectures and desired properties. In fact, combining nanoparticles with different physical and chemical properties offers a large number of possibilities, making it possible to tailor the properties of agglomerated materials. In addition to the specific properties of the individual nanoparticle, their assembly gives rise to collective properties due to nanoparticle interactions. Compared to the bulk materials, this new configuration is more flexible for material preparation. For example, it allows for the combination of different and even conflicting properties—such as large submicrometer-sized particles that do not lose their large surface area at nanoscale.

The agglomeration of nanoparticles is a consequence of nanoparticle destabilization, and it depends on both the nanoparticles’ properties (size, shape, surface modification, and concentration) and embedding media. In aqueous solution, the agglomeration behavior of anatase TiO_2_ nanoparticles is predicted by DLVO (Derjaguin, Landau, Vervey and Overbeek) theory [1], which shows that with the increase of nanoparticles’ size, both their energetic barrier and their critical concentration of agglomeration become higher. On the other hand, small nanoparticles favor agglomeration because of their lower energetic barrier. A linear behavior was reported between particle size and critical concentration—from which their agglomeration takes place [2,3]. In fact, nanoparticle agglomeration is controlled by electrostatic (repulsive) and van der Waals (attractive) interactions, according to DLVO theory [4,5,6]. When electrostatic repulsion dominates the interaction between nanoparticles, the aggregation occurs at a lower rate. However, when the electrostatic repulsion is eliminated because of charge screening at the nanoparticle surfaces, the attractive (van der Waals) interactions dominate and most of the nanoparticle collisions induce their aggregation. From DLVO theory, it is clear that to induce nanoparticle agglomeration, it is necessary to reduce repulsive interaction and/or to enhance the collision speed between nanoparticles.

Within the morphologies and materials studied, TiO_2_ nanostructured materials have received particular attention due to their use as photoanodes for potential application in many areas, including photovoltaics [7], photo catalysis [8], gas sensing [9], photocatalytic water splitting [10] and Li-ion batteries (LiB) [11,12]. The advantages of TiO_2_-based materials used as negative electrodes in LiB are: low cost, very good structural stability during cycling, very small volume changes (~4%), good capacity retention and fast Li kinetics [13,14,15,16]. Drawbacks of TiO_2_ include low (180 mAh/g) reversible capacity (with reference to carbonaceous negative electrodes, which are 372 mAh/g), poor electrical conductivity and a low ionic diffusivity. 

The diffusion coefficient and diffusion lengths depend on the nature of the materials and the particle size, respectively. Different nanostructured TiO_2_ materials have been thoroughly investigated, including nanoparticles, nanowires, nanotubes, nanorods and nanoneedles [17,18,19,20]. In the case of electrode materials, several other parameters must be taken into account to optimize the performance of LiBs, including chemical composition, material structure and architecture [21]. It has been demonstrated that the electrochemical performance of porous electrode materials depends strongly on pore properties (size, distribution and tortuosity) [22]. An optimal anode material must satisfy the following requirements: a large specific surface area in contact with the electrolyte (to ensure large Li-ion insertion), low volume changes of electrode material during the insertion/desertion process (crucial for better cycling stability), and fast Li diffusion within the electrode material. These factors depend on pore properties [23].

In this work, TiO_2_ mesoporous aggregates made using TiO_2_ nanoparticles as a building unit were successfully prepared by hydrothermal synthesis. It was shown that the size of prepared TiO_2_ aggregates depended on the synthesis temperature. The TiO_2_ anode performances in lithium-ion batteries was discussed in terms of the conflicting effects of the specific surface area and the pore properties of the TiO_2_ aggregates used.

## 2. Materials and Methods

### 2.1. Synthesis of TiO_2_ Nanoparticles and Aggregates

For the hydrothermal synthesis of TiO_2_ nanoparticles, titanium (IV) oxysulfate hydrate (TiOSO_4_, Sigma Aldrich, Saint-Quentin-Fallavier, France) and titanium tetrachloride (TiCL_4_, Fluka, Saint-Quentin-Fallavier, France) precursors were used. All the chemicals were analytical grade, and used without further purification. The TiOSO_4_ precursor solution was prepared by dissolving 6.4 g of TiOSO_4_ (2.5 M) in 16 mL of distilled water (Milli Q System, electric resistivity 18.2 MΩ·cm, Merck, St Quentin en Yvelines, France) under constant stirring at 750 r/min and a temperature of 45 °C for 2 h to get a clear solution. Then, the solution of TiOSO_4_ was transferred into a Teflon-lined stainless steel autoclave with a 25 mL capacity. The heating rate was 2.5 °C/min. During synthesis, the temperature was maintained at different temperatures—100, 150, 180, 190, 200 and 220 °C—for 6 h, depending on the aggregate size required. After synthesis in autoclave, a white TiO_2_ powder was obtained, then washed 6 times in distilled water and 2 times in ethanol. Finally, the obtained powder was dried overnight in the oven and annealed in air at 500 °C for 30 min with the heating rate at 5 °C/min.

### 2.2. Characterization of Prepared of TiO_2_ Nanoparticles and Aggregates

The morphological investigations of prepared of TiO_2_ nanoparticles and aggregates were achieved with a high-resolution Ultra 55 Zeiss FEG scanning electron microscope (FEGSEM, Zeiss, Marly Le Roi., France) operating at an acceleration voltage of 10 kV.

The crystalline structure of TiO_2_ was determined by an X-ray diffractometer (Siemens D5000 XRD unit, Siemens, Toulouse, France) in 2θ range from 20° to 80° by 0.07° s^−1^ increasing steps, operating at 40 kV accelerating voltage and 40 mA current, using a Cu Kα radiation source with λ = 1.5406 Å.

Nitrogen adsorption-desorption isotherms were measured at liquid nitrogen temperature on a Micromeritics ASAP 2020 apparatus (Micromeritics, Mérignac, France). Before analysis, all the samples were degassed at 120 °C for 10 h. The specific surface area (SBET) was evaluated using the Brunauer-Emmett-Teller (BET) method in the P/P° range of 0.05–0.25. The pore size distribution was determined from the desorption branch of the isotherm using the Barret-Joyner-Halender (BJH) method. The total pore volume was determined from the amount of N_2_ adsorbed up to P/P° = 0.98.

The chemical compositions of all the samples (TiO_2_ aggregates, nanoparticles and aggregates) were determined by X-ray photoelectron spectroscopy (XPS) using a Thermos K-Alpha system equipped with an Al Kα X-ray source (hυ = 1486.6 eV; spot size = 400 μm).

The electrochemical tests were performed in Teflon Swagelok half-cells. A TiO_2_ composite electrode was used as a working electrode, and a Li metal foil (Sigma Aldrich) was used as a reference and counter electrode in a battery-grade electrolyte (1M LiPF_6_ ethylene carbonate (EC), propylene carbonate and dimethyl carbonate (DMC)), 1:1:3, *v/v/v* with 1% wt vinylene carbonate, purchased from Solvionic, Toulouse, France). The TiO_2_ composite electrode was prepared by mixture of our TiO_2_ active material (80 wt.%) with 7 wt.% of mesoporous carbon, 7 wt.% graphite powder and 6 wt.% polytetrafluoroethylene (PTFE). A glass microfiber filter (Grade GF/D (Whatman)) 0.67 mm thick with a pore size of 2.7 μm (GmF) was used as a separator. The Swagelok cells were assembled in the MBraun Glove Box (with H_2_O < 1 ppm and O_2_ < 1 ppm, MBraun, Mérignac, France). Assembled batteries were galvanostatically cycled in the voltage range between 3–1.0 V vs. Li/Li^+^ at a charge/discharge rate of C/10 (full charge or discharge in 10 h), using a VMP3 Biologic multichannel potentiostat/galvanostat (Biologic, Seyssinet-Pariset, France).

## 3. Results

### 3.1. Synthesis, Characterization and Formation Mechanism of TiO_2_ Nanoparticle Aggregates

**Figure 1:**
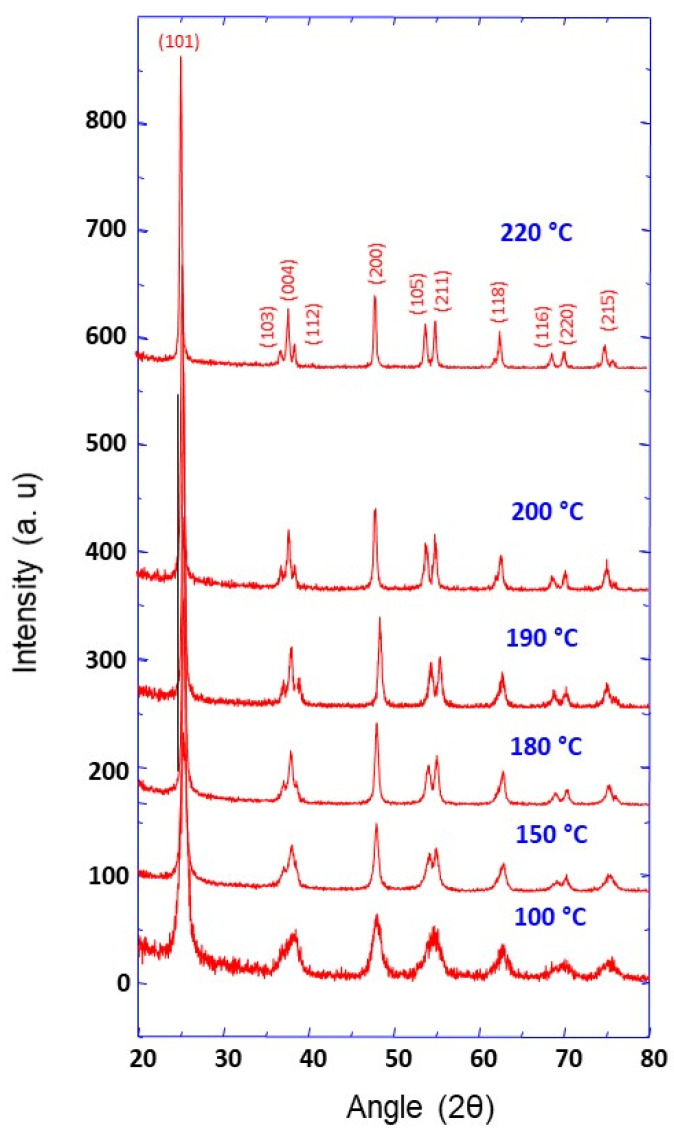
XRD pattern of TiO_2_ nanoparticle aggregates prepared at different synthesis temperatures as indicated.

The obtained white TiO_2_ powder was characterized using different techniques. The crystalline structure and phase of the TiO_2_ powder (prepared at different temperatures of about 100, 150, 180, 190, 200 and 220 °C) were investigated by X-ray diffraction. The obtained XRD patterns, presented in Figure 1, reveal a crystalline structure with all the peaks assigned to the TiO_2_ anatase phase (JCPDS No. 89-4921), indicating high-purity material. It can also be observed that the peaks’ resolution is enhanced with the synthesis temperature, which can be attributed to crystallinity improvement.

**Figure 2:**
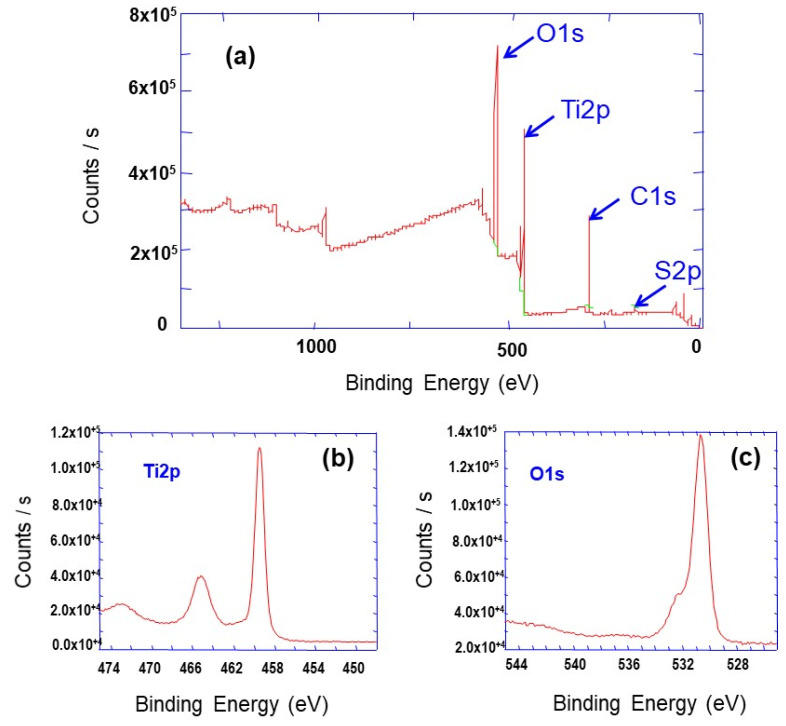
XPS spectra of TiO_2_ prepared aggregates prepared at synthesis temperature of 200 °C (**a**) XPS survey (**b**) Ti2p core level (**c**) O1s core level.

In addition, the average crystallite sizes were calculated using Scherrer’s analysis of the full width at half-maximum of the intense peak, corresponding to the (101) crystallographic plane (Table 1). For the synthesis temperatures mentioned above, calculated TiO_2_ nanoparticles sizes were found to be about 9.8, 17.9, 20.7, 20.7, 24.7 and 30.4 nm, respectively. The nanoparticles’ size increased with the increase of synthesis temperature. Furthermore, the chemical composition of the prepared powder was analyzed by XPS; the obtained spectra are depicted in Figure 2. The XPS survey spectrum in Figure 2a shows the intense peaks, corresponding to O1s and Ti2p core levels. The high-resolution XPS spectra in Figure 2b shows two oxygen peaks: an intense O1s peak located at 529.7 eV (corresponding to O in Ti-O lattice) and a higher binding energy peak at 532.5 eV attributed to adsorbed water and/or O−C=O [24]. The Ti2p peaks (Figure 2c), located at 464.1 and 458.4 eV, correspond to the spin orbit doublet of Ti2p (Ti2p_1/2_ and Ti2p_3/2_, respectively) and can be attributed to TiO_2_ [25,26]. The atomic percentage ratio of oxygen (O1s at 529.7 eV with 38.6 at %) to titanium (14.6 at %) for the as-prepared powder shows that the O to Ti ratio was slightly higher than theoretically expected (2). This discrepancy can be ascribed to surface adsorption of different species and/or to the presence of oxygen vacancies in TiO_2_ [27].

**Figure 3:**
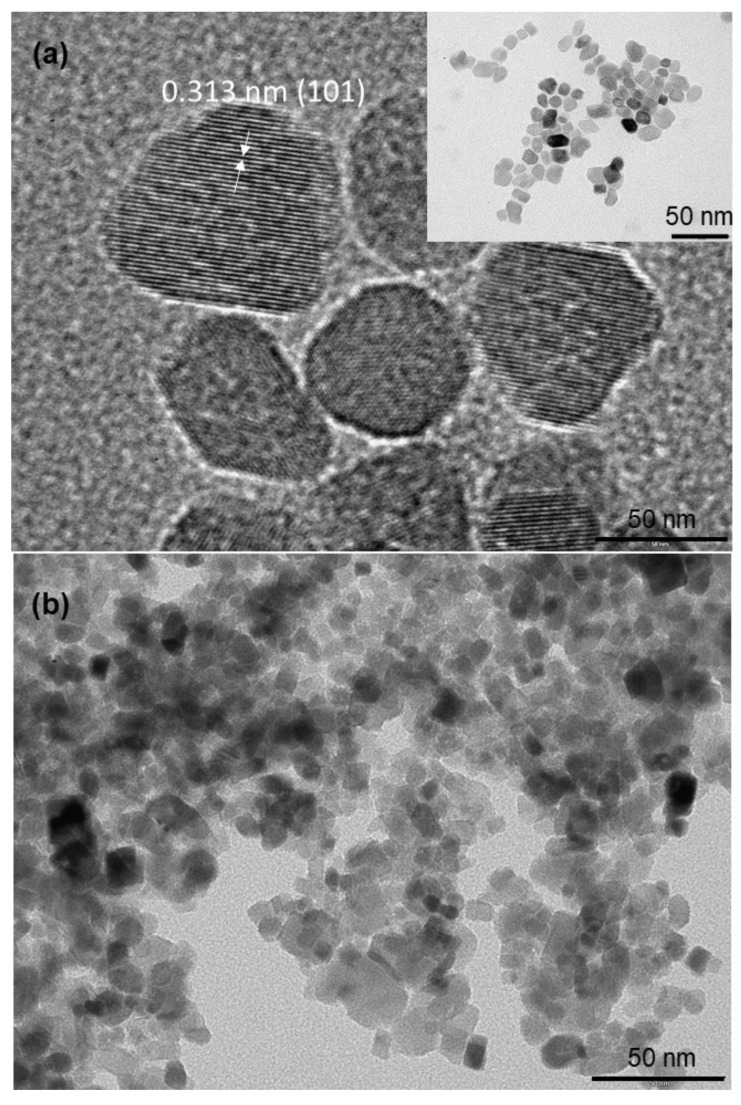
TEM images of TiO_2_ nanoparticle synthesis at different synthesis temperatures (**a**) HRTEM of nanoparticles obtained at synthesis temperature of 220 °C; inset shows the corresponding high magnification (**b**) nanoparticles obtained at synthesis temperature of 150 °C; similar morphology was observed for 180, 190 and 100 °C.

The crystallinity and morphology of the prepared TiO_2_ nanoparticles were investigated by means of HRTEM (high-resolution transmission electron microscopy) and transmission electron microscopy (TEM), respectively; the results are depicted in Figure 3. Figure 3a shows crystal lattice planes for TiO_2_ prepared at 220 °C with isolated nanoparticles, as demonstrated in the insert. In addition, the measured distance (d) of interlattice planes was about 0.301 nm, corresponding to a distance between (110) crystallographic planes of TiO_2_ in anatase phase. For lower temperatures, TEM patterns in Figure 3b show aggregates formed by smaller coalesced nanoparticles, with sizes ranging from 10 to 30 nm.

**Figure 4:**
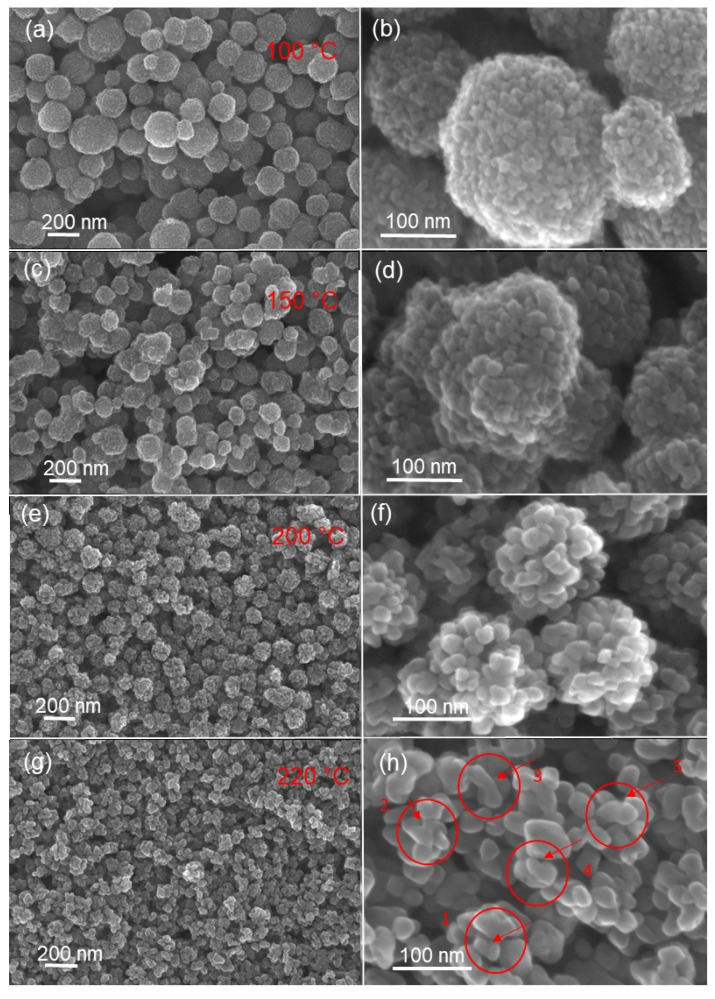
FEGSEM images of TiO_2_ aggregates obtained at different synthesis temperatures: (**a**) 100 °C, (**c**) 150 °C, (**e**) 200 °C and (**g**) 220 °C. (**b**,**d**,**f**,**h**) show, respectively, the corresponding high magnifications.

**Figure 5:**
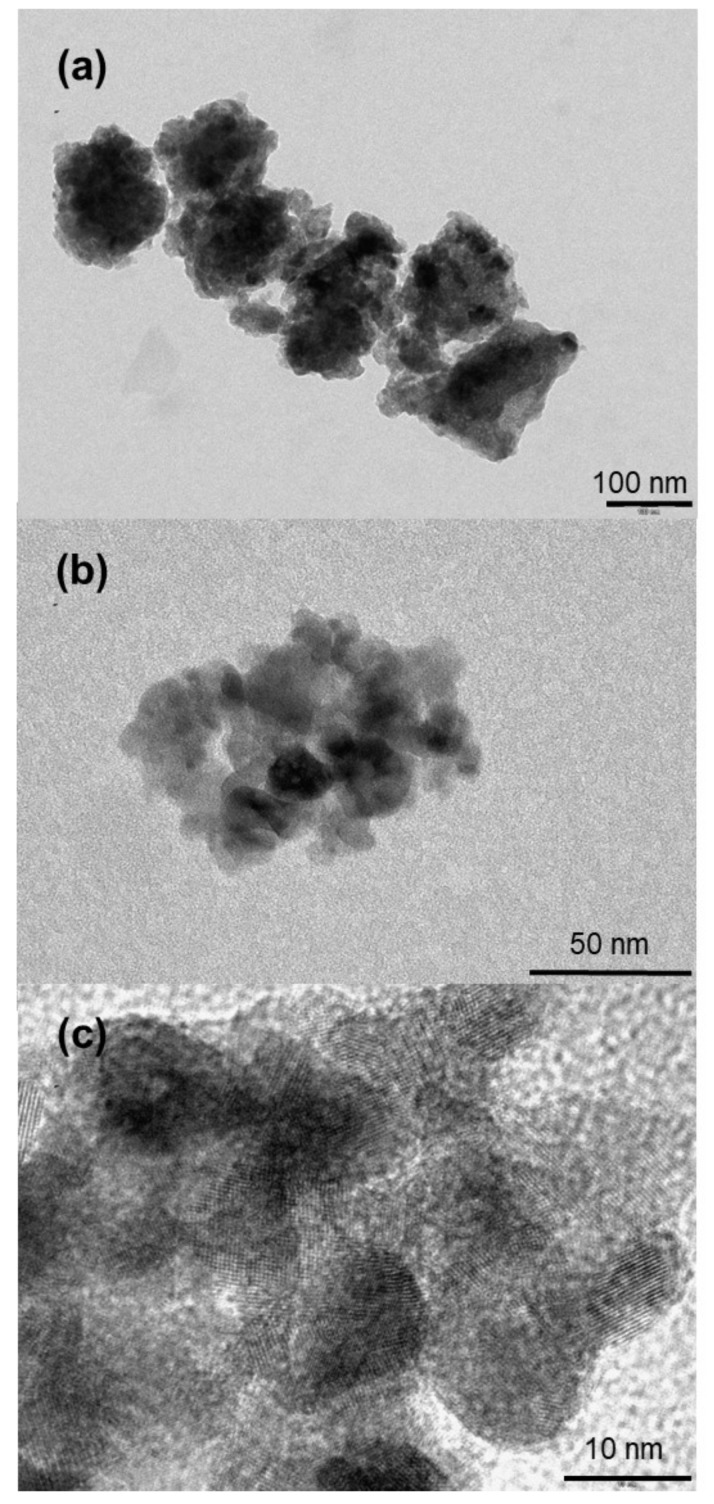
TEM images at different magnifications of TiO_2_ aggregates (**a**–**c**) prepared at synthesis temperature of 200 °C.

The particles were more or less faceted. The morphology of the prepared TiO_2_ aggregate was characterized by FEGSEM (Figure 4). The aggregates were more or less spherical, with sizes ranging from 10 to 200 nm. The aggregates were made of polydisperse TiO_2_ nanoparticles, whose size increased with the synthesis temperature as shown in Figure 4b,d,f,h and Figure 5a,b.

**Figure 6:**
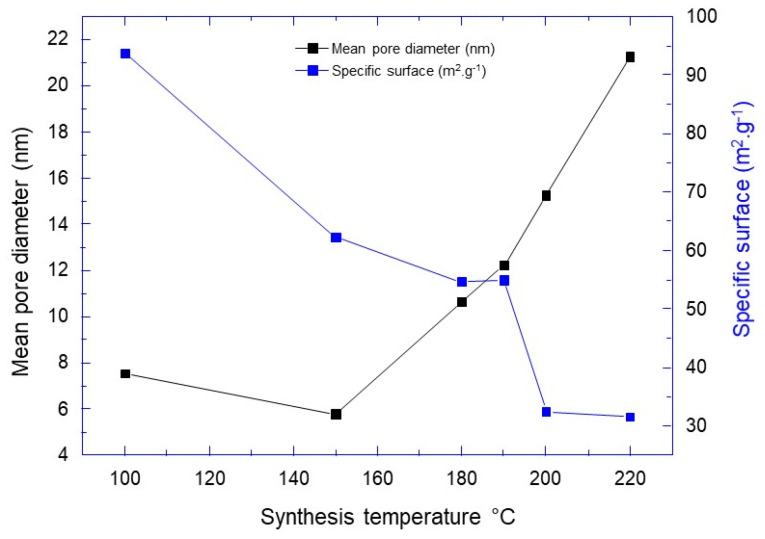
Variation of aggregate characteristics in terms of mean pore diameter and specific surface versus synthesis temperature.

The specific surface area and the average pore size of the prepared TiO_2_ aggregates were subsequently estimated using the Brunauer-Emmett-Teller method, through the nitrogen adsorption/desorption isotherms. From the hysteresis loop of the nitrogen isotherm curves, the corresponding BET-specific surface areas were calculated to be 93.78, 62.29, 54.66, 54.98, 32.51 and 31.62 m^2^·g^−1^ for synthesis temperatures of 100, 150, 180, 190, 200 and 220 °C, respectively (Figure 6 and Table 1).

Based on the Barrett-Joyner-Halenda (BJH) method and the desorption branch of the nitrogen isotherms, the average calculated pore size was about 7.53, 5.79, 10.64, 12.24, 15.26 and 21.28 nm, respectively, for the same synthesis temperatures mentioned above (Figure 6 and Table 1). The BET and BJH characteristics of porous TiO_2_ aggregates were in agreement with the nanoparticle sizes obtained from DRX experiments, showing the increase of TiO_2_ nanoparticle size, the pore size of TiO_2_ aggregates and the specific surface decreases. From these results, it is clear that high specific surface and large pore size are conflicting requirements. The large surface areas enable high exposed surface, large pore size favors fast diffusion and transfer toward the material surface. A balance between these two properties is needed to optimize the material.

The controlled assembly of TiO_2_ nanoparticles into aggregates of spherical shape and controlled size was demonstrated to depend strongly on the synthesis temperature. Furthermore, it was demonstrated that, when the temperature increased, the aggregate size decreased. This may raise questions about the mechanism of the nanoparticle aggregates’ formation. In fact, the aggregation rate of nanoparticles depends on a combination of various parameters, related to the solvent, nanoparticle properties and synthesis parameters of the hydrothermal method.

In traditional crystal growth, theories generally postulate that crystals grow by addition of individual atoms or ions into an interfacial region. However, individual atoms are reduced to form nuclei, which grow into small particles through aggregation and coalescence. The growth rate of the particle diameter slows down as a depletion layer of atoms takes place around the particles. When particles grow larger and their growth rate slows, their self-assembly becomes predominant. During the packing process, particles coagulate and rotate with respect to each other until their crystal orientation facets coincide [28].

In general, small nanoparticles favor agglomeration because of the lower energetic barrier. A linear behavior was reported between particle size and critical concentration, from which their agglomeration takes place [2,3]. Nanoparticle agglomeration is controlled by electrostatic (repulsive) and van der Waals (attractive) interactions, according to DLVO theory [4,5,6]. When the electrostatic repulsion dominates the interaction between nanoparticles, the aggregation slows down. However, when the electrostatic repulsion is eliminated because of the charge screening at the nanoparticles’ surfaces, the attractive van der Waals interactions dominate, and most of the nanoparticles’ collisions induce their aggregation. From DLVO theory, it is clear that to induce nanoparticles agglomeration, it is necessary to reduce repulsive interaction and/or to enhance the collision rate between nanoparticles.

On the other hand, for small nanoparticles, the effect of gravity can be ignored; the stability of nanoparticles is ensured by a combination of van der Waals attraction, steric and electrostatic forces. In addition, due to thermal agitation, a diffusion counteracts nanoparticle sedimentation, but induces collisions between nanoparticles. However, the rate of collisions and subsequent coalescence do govern the size of the particle aggregates. In fact, there is a competition between nanoparticle collisions and their coalescence, though not all the collisions lead to aggregation and/or coalescence. At high temperatures (and thus high thermal agitation) nanoparticle velocity is higher, which induces dissipation of kinetic energy through nanoparticle deformation; any excess energy is converted into kinetic energy of the nanoparticle rebound. Now, if the adhesion between nanoparticles is greater than the exceeding kinetic energy, nanoparticles form aggregates. In the opposite case, lower temperature leads to lower nanoparticle velocities, in which kinetic energy is dissipated, mainly through nanoparticle deformation. Additionally, high deformation-prone nanoparticles adhesion and thus their aggregation [29]. It is well known that there is a correlation between surface charge and nanoparticle size in metal oxides. In fact, it was reported that the point of zero charge (PZC) shifts toward a lower pH value as nanoparticle size increases for a similar aspect ratio [30,31,32]. In addition, when the nanoparticles’ size decreases, it may approach that of the counter ions, which could provoke the charge screening of the nanoparticle half-surface and, in turn, favor the nanoparticles’ coalescence.

In a self-assembly process without any energy barriers, the probability of a nanoparticle attaching to a certain facet of another particle is roughly proportional to the area of this facet [33]. Particles follow different mechanisms to self-assemble, based on their properties in terms of size and morphology. Based on these different mechanisms of self-assembly, different compactnesses of their assembly could be obtained. Rather than individual atoms, particles are the basic building blocks, and form aggregate structures. In Figure 3a,b it can be observed that the more particles are faceted, the more the structure of their assembly could be compact—with less void in the formed structure.

In light of the presented bibliographic data for similar nanoparticle sizes, including those obtained here in this study, it can be proposed that higher temperatures increase solvent fluctuations and, in turn, Brownian motion. This leads to enhancement in nuclei collisions, favoring their agglomeration and coalescence, to form (in the later stage) large nanoparticles with solubility, at which point no additional nanoparticles are formed. At a later stage, the agglomeration of these larger nanoparticles takes place; the aggregate size is governed by the different parameters discussed above and could be summarized in the repulsive interactions and the collisions between nanoparticles.

In the case of large nanoparticles, the repulsive interactions are higher because of the high charge on their surface, which favors the formation of small-sized prepared aggregates (Figure 4g,h). Conversely, at low temperatures, the agglomeration and coalescence of nuclei is not favored, and small nanoparticles are formed. Furthermore, the repulsive interactions between small nanoparticles are lower, due to the small charges on their surfaces. This favors their adhesion and agglomeration, explaining the formation of large aggregates (Figure 4a,b). It is clear that to induce nanoparticle agglomeration, it is necessary to reduce the repulsive interaction and/or to enhance the collision rate between nanoparticles.

A closer analysis of FEGSEM (Figure 4) shows different steps of the coalescence process. The first step was nanoparticle contact (region 1, indicated in Figure 4f). Later, the coalescence process began with the formation of necks between the joined nanoparticles (region 2–4, indicated in Figure 4h). The evolution of formed necks is driven by surface atom diffusion to minimize surface energy [34,35,36]. In Figure 4, region 1–5, it can be observed that after the formation of necks between joined nanoparticles, they disappear through the formation of larger particles (region 5, indicated in Figure 4h). However, the formation of these larger particles enhances pore size. This coalescence process explains the fact that, for the synthesis temperatures of 180 °C and 190 °C, the obtained powders showed the same specific surface; it also explains the increase of pore size as temperature increases. The same behavior is observed for synthesis temperatures of 200 °C and 220 °C. It is well-known that pore size increases with nanoparticle size; however, this was not the case in our experiments. In the present results, nanoparticle coalescence could explain the observed peculiarity.

### 3.2. Electrochemical Test of TiO_2_ Nanoparticle Aggregates in LiB

An understanding of nanoparticle aggregation mechanisms and the parameters controlling their specific surface and porosity was necessary for the application and enhancement of their properties as negative electrode material for LiB. Before discussing the influence of nanoparticle aggregate architecture, the influence of nanoparticle properties will be discussed, in terms of their performances as potential negative electrodes for LiB.

**Figure 7:**
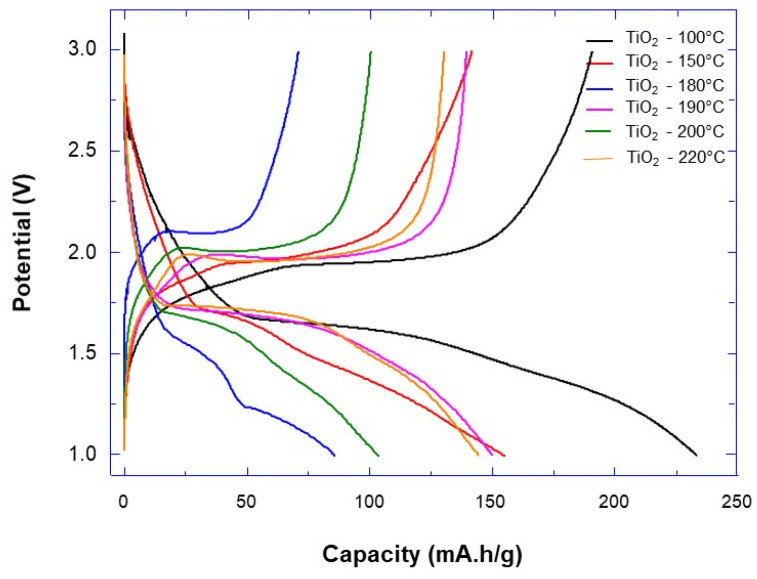
Galvanostatic charge-discharge curves at C/10 rate in the voltage range between 3–1.0 V vs. Li/Li^+^ for TiO_2_ aggregates prepared at different synthesis temperatures.

The discharge/charge curves recorded for TiO_2_ powder samples prepared at different synthesis temperatures are presented in Figure 7. It can be clearly observed that the specific capacity increased as a function of the decreasing synthesis temperature. The capacity of discharge was always higher than the capacity of charge, indicating some irreversible capacity resulting from the surface reactivity of TiO_2_ nanoparticles with electrolyte reduction and the formation of a solid electrolyte interphase (SEI) layer. The highest capacity of about 233 mAh/g was observed for the initial discharge process for the TiO_2_ synthesized at 100 °C. This capacity was higher than the capacity of 168 mAh/g [37,38] usually obtained for TiO_2_ and corresponded to the insertion of 0.5 Li into TiO_2_, according to the following electrochemical equation:

TiO_2_ + 0.5Li^+^ + 0.5e^−^ → Li_0.5_TiO_2_.

For the samples synthetized at 100 °C, the observed higher capacity corresponded to the insertion of about 0.7 Li_0.7_TiO_2_, which may be ascribed to the nanoscale of TiO_2_ nanoparticles forming the aggregate—providing a high contact surface area between the electrolyte and the electrode surface and a short diffusion path for both the electron and the Li ion [21].

Furthermore, it has been reported that for bulk anatase TiO_2_, the curves (discharge/charge) are characterized by a plateau at 1.8 V, which can be attributed to a two-phase Li insertion/desertion process into the TiO_2_ lattice [39]. Our results showed that the plateau at 1.7 V was shorter, compared to that of the bulk TiO_2_. This kind of behavior was observed and explained by the fact that the powder behaves as a solid solution [40]. The length and potential of the plateau varied as a function of the synthesis temperature. The decrease in potential was observed for temperatures from 100 to 180 °C, while a stable potential plateau was observed for temperatures 190 to 220 °C. The capacity of discharge also changed as a function of the synthesis temperature. It was previously reported that the nanoparticles’ crystallinity played a crucial role in the resulting specific capacity. The high crystallinity of TiO_2_ anatase reduced the diffusion length of the Li ion to adjacent sites [41,42,43]. In addition, the increasing of the crystallinity reduced the oxygen vacancy defects, which in turn favored the surface desorption of hydroxyl groups and water [44]. This improved the Li ion’s ability to reach the surface and be inserted in the electrode material lattice. It should be also noted that a desorption of hydroxyl groups and water could lead to the formation of less significant SEI layers than in the presence of some adsorbed hydroxyl groups or water on the surface of TiO_2_ nanoparticles [45].

The formation of less important SEI layers on the samples synthetized at higher temperatures (≥150 °C) could be explained by the much lower irreversible capacity (of around 6% ± 2%) than that obtained for the sample synthetized at 100 °C (around 30%).

Herein, we will discuss how control over the nanoparticle aggregates’ properties may be used to improve the performance of Li-ion batteries. It has been clearly demonstrated here that pore size plays a major role in the optimization of the Li-ion battery’s specific capacity. With synthesis temperatures of 180 and 190 °C, which have similar specific surface, the decrease in pore size enabled the specific capacity improvement. Similar results were observed with synthesis temperatures of 200 and 220 °C (Table 1).

As was mentioned previously, nanoparticle aggregates are porous materials whose architecture is characterized by porosity, tortuosity and specific surface. However, it is quite evident that these parameters also play a key role in the diffusion of Li ions within battery electrodes. Therefore, it is essential to mention that these parameters are dependent and behave in conflicting ways. An increase in pore size will enhance ion diffusion within the electrode material, but will also lower the specific surface. A good balance between pore size and specific surface must be found to optimize the performance of Li-ion batteries.

It was previously reported that some mesopores and micropores play a minor role in specific capacity enhancement, due to pore-clogging by some reaction products [23], which in consequence reduce the specific surface and lead to lower specific capacity. The same results were observed for our samples, as reported in Table 1. Samples with synthesis temperatures of 100 and 150 °C exhibited specific capacity decreases along with the pore size. This result was very surprising, if we take into account the fact that reduction of the pore size should induce the enhancement of the specific surface, and in turn the specific capacity, as previously observed by Lin et al. [23]. However, it is possible that these small pores are more likely to be blocked by side reaction products (e.g., electrolyte reduction or solvated ions). This would result in a reduction of the specific surface and reactivity at the electrode/electrolyte interface, which in turn would lead to a lower specific capacity. Similarly, it was previously observed that capacity loss was related to electrolyte reduction at the pores’ wall [46].

The highest capacity was obtained for the sample prepared at 100 °C, with a higher specific surface and intermediate pore size compared to other samples. This could be explained by the high specific surface and the presence of large pores even after deposition of reaction products at electrodes on their walls. In that case, there should be a balance or equilibrium between the specific surface and pore size reduction to keep the highest specific capacity of the LiB.

In addition, the pore connectivity should have an important impact on the optimization of energy storage of porous electrodes [47]. However, in the present study, due to nanoparticle shape similarity and their assembly in spherical aggregates, we assume that the connectivity would be similar for all prepared powders. In addition, as TiO_2_ is known to have low volume change during the insertion/desertion process, it is reasonable to conclude that pore connectivity may be similar for all prepared powders.

## 4. Conclusions

A synthesis of spherical TiO_2_ aggregates of controlled diameter and porosity was achieved using the hydrothermal method. This method can be applied in large-scale production of TiO_2_ electrode materials. It was demonstrated that the TiO_2_ aggregates were made of TiO_2_ nanoparticles as a building unit. The increase of synthesis temperature favored the increase of TiO_2_ nanoparticle size and the decrease of its formed aggregate diameter. The as-prepared TiO_2_ aggregate powder was tested as a potential negative electrode material for Li-ion batteries. The results showed clearly that both the aggregate specific surface and pore size played a major role in optimizing the specific capacity of TiO_2_ negative electrode materials. It was demonstrated for the TiO_2_ powders with similar specific surface that the pore size decrease lowered the specific capacity. This was explained by a possible pore-clogging by products of electrolyte reduction, which in turn reduced the electrode specific surface. Furthermore, the highest specific capacity was observed for the TiO_2_ powder prepared at 100 °C, which provided a balance between pore size and specific area. The high specific surface ensured a good electrode/electrolyte interface, and the large pore size enhanced the Li ion diffusion. The coalescence process explained the fact that, for synthesis temperatures of 180 and 190 °C, the obtained powders showed the same specific surface. It also explained the increase in pore size as the temperature increased. In addition, the same behavior was observed for synthesis temperatures of 200 and 220 °C. The well-known behavior of the pore size increasing along with the nanoparticle size was not observed in the case of the powders prepared at synthesis temperatures of 200 and 220 °C. This peculiarity was explained by the nanoparticles’ coalescence.

## Figures and Tables

**Table 1:** TiO_2_ aggregate characteristics synthesized at different temperatures and their electrochemical performance as negative electrode materials.

Synthesis Temperature (°C)	Crystallite Size (nm)	Mean Pore Diameter (nm)	Specific Surface (m^2^·g^−1^)	Capacity Discharge/Charge (mA·h/g)
100	9.8	7.53	93.78	233/190
150	17.9	5.79	62.29	155/142
180	20.7	10.64	54.66	71/86
190	20.7	12.24	54.98	139/150
200	24.7	15.26	32.51	100/104
220	30.4	21.28	31.62	144/136

## Data Availability

The data presented in this study are available on request from the corresponding author.

## References

[1] Cao T., Trefalt G., Borkovec M. (2018). Aggregation of colloidal particles in the presence of hydrophobic anions: Importance of attractive non-DLVO forces. Langmuir.

[2] He Y.T., Wan J., Tokunaga T. (2007). Kinetic stability hematic nanoparticles the effect of particle size. J. Nanoparticle Res..

[3] Zhou D., Ji Z., Jiang X., Dunphy D.R., Brinker J., Keller A. (2013). Influence of material properties on TiO_2_ nanoparticle agglomeration. PLoS ONE.

[4] Derjaguin B. (1939). A theory of interaction of particles in presence of electric double-layers and the stability of lyophobe colloids and disperse systems. Acta Phys. Chem..

[5] Derjaguin B., Landau L.D. (1941). Theory of the stability of strongly charged lyophobic sols and of the adhesion of strongly charged particles in solutions of electrolytes. Acta Phys. Chim..

[6] Verwey E.J.W., Overbeek J.T.G. (1948). Theory of Stability of Lyophobic Colloids.

[7] Tan B., Wu Y.Y. (2006). Dye-sensitized solar cells based on anatase TiO_2_ nanoparticles/nanowires composites. J. Phys. Chem. B.

[8] Han C., Luque R., Dionysiou D. (2012). Facile preparation of controllable size monodisperse anatase titaniananoparticles. Chem. Comm..

[9] Maziarz W., Kusior A., Trenczek-Zajac A. (2016). Nanostructured TiO_2_-based gas sensors with enhanced sensitivity to reducing gases. Beilstein J. Nanotechnol..

[10] Sun B., Shi T., Peng Z., Sheng W., Jiang T., Liao G. (2013). 2013 Controlled fabrication of Sn/TiO_2_ nanorods for photoelectrochemical watersplitting. Nanoscale Res. Lett..

[11] Yan X., Wang Z., He M., Chen X. (2015). TiO_2_ nanomaterials as anode materials for lithium-ion rechargeable batteries. Energy Technol..

[12] Sugiawati V., Vacandio F., Galeyeva A., Kurbatov A., Djenizian T. (2019). Enhanced electrochemical performance of electropolymerized self-organized TiO_2_ nanotubes fabricated by anodization of Ti grid. Front. Phys. Front..

[13] Li X., Zhang Y., Li T., Zhong Q., Li H., Huang J. (2014). 2014 Graphene nanoscrolls encapsulated TiO_2_ nanowires for lithium storage. J. Power Sources.

[14] Jin J., Huang S.Z., Liu J., Li Y., Chen D.S., Wang H.E., Yu Y., Chen L.H., Su B.L. (2014). Design of new anode materials based on hierarchical three-dimensional ordered macro mesoporous TiO_2_ for high performance lithium-ion batteries. J. Mater. Chem. A.

[15] Wagemaker M., Kearley G.J., van Well A.A., Mutka H., Mulder F.M. (2003). Multiple Li positions inside oxygen octahedra in lithiated TiO_2_ anatase. J. Am. Chem. Soc..

[16] Xie M., Sun X., Zhou C., Cavanagh A.S., Sun H., Hu T., Wang G., Lian J., George S.M. (2015). Amorphous Ultrathin TiO_2_ atomic layer deposition films on carbon nanotubes as anodes for lithium-ion batteries. J. Electrochem. Soc..

[17] Liu Y., Yang Y. (2016). Recent progress of TiO_2_-based anodes for Li ion batteries. J. Nanomater..

[18] Jiang C., Zhang J. (2013). Nanoengineering titania for high rate lithium storage: A review. J. Mater. Sci. Technol..

[19] Zhang Y., Tang Y., Li W., Chen X. (2016). Nanostructured TiO_2_-based anode materials for high-performance rechargeable lithium-ion batteries. Chem. Nano Mat..

[20] Liu Z., Andreev Y.G., Armstrong A.R., Brutti S., Ren Y., Bruce P.G. (2013). Nanostructured TiO_2_(B): The effect of size and shape on anode properties for Li-ion batteries. Prog. Nat. Sci. Mater. Int..

[21] Tarascon J.M., Armand M. (2001). Issues and challenges facing rechargeable lithium batteries. Nature.

[22] Li J.M., Wan W., Zhou H.H., Li J.J., Xu D.S. (2011). Hydrothermal synthesis of TiO_2_(B) nanowires with ultrahigh surface area and their fast charging and discharging properties in Li-ion batteries. Chem. Commun..

[23] Lin Z., Zheng M., Zhao B., Wang G., Pu L., Shi Y. (2014). Influence of the pore structure parameters of mesoporous anatase microspheres on their performance in lithium-ion batteries. J. Solid State Electrochem..

[24] Swiatowska-Mrowiecka J., Zanna S., Ogle K., Marcus P. (2008). Adsorption of 1,2-diaminoethane on ZnO thin films from p-xylene. Appl.Surf. Sci..

[25] Gadois C., Światowska J., Zanna S., Marcus P. (2013). Influence of titanium surface treatment on adsorption of primary amines. J. Phys. Chem. C.

[26] Ma J., Li W., Morgan B.J., Światowska J., Baddour-Hadjean R., Body M., Legein C., Borkiewicz O.J., Leclerc S., Groult H. (2018). Lithium intercalation in anatase titanium vacancies and the role of local anionic environment. Chem. Mater.

[27] Ennaceri H., Boujnah M., Taleb A., Khaldoun A., Köhler T., Sáez-Araoz R., Ennaoui A., Benyoussef A. (2017). Thickness effect on the optical properties of TiO_2_-anatase thin films prepared by spray-ILGAR: Experimental and ab initio studies. Int. J. Hydrog. Energy.

[28] You H., Chen F., Yang S., Yang A., Ding B., Liang S., Song X. (2011). Size effect on nanoparticles mediated silver crystal growth. Cryst. Growth Des..

[29] Hawa T., Zachariah M.R. (2004). Molecular dynamic study of particle-particle collisions between hydrogen-passivated silicon nanoparticles. Phys. Rev. B.

[30] Liu X., Chen G., Su C. (2011). Effect of material properties on sedimentation and aggregation of TiO_2_ nanoparticles of anatase and rutile in the aqueous phase. J. Colloid. Interf. Sci..

[31] Suttiponparnit K., Jiang J., Sahu M., Suvachittanont S., Charinpanitkul T., Biswas P. (2010). Role of surface area, primary particle size and crystal phase on titanium dioxide nanoparticles dispersion properties. Nanoscale Res. Lett..

[32] Abbas Z., Labbez C., Nordholm S., Ahlberg E. (2008). Size-dependent surface charging of nanoparticles. J. Phys. Chem. C.

[33] Zhou Q., Wang B., Wang P., Dellago C., Wang Y., Fang Y. (2013). Nanoparticle-based crystal growth via multistep self-assembly. Cryst. Eng. Comm..

[34] Lim T.H., McCarthy D., Hendy S.C., Stevens K.J., Bishop S.A., Tilley R.D. (2009). Real-time TEM and kinetic monte carlo studies of the coalescence of decahedral gold nanoparticles. ACS Nano.

[35] Ingham B., Lim T.H., Dotsler C.J., Henning A., Toney M.F., Tilley R.D. (2011). How nanoparticles Coalesce: An in-situ study of Au nanoparticle aggregation and grain growth. Chem. Mater..

[36] Kohn P., Pathak S., Stefik M., Ducati C., Wiesner U., Steiner U., Guldin S. (2013). Low temperature crystalisation of mesoporous TiO_2_. Nanoscale.

[37] Subramanian V., Karki A., Gnanasekar K.L., Eddy F.P., Rambabu B. (2006). Nanocrysatlline TiO_2_ (anatase) for Li ion batteries. J. Power Sources.

[38] Krtil P., Fattakhova D., Kavan L., Burnside S., Gratzel M. (2000). Lithium insertion into self-organized mesoscopic TiO_2_ electrodes. Solide State Ion..

[39] Wagemaker M., Borghols W.J.H., Mulder F.M. (2007). Large impact of particle size on insertion reaction. A case for anatase Li*_x_*TiO_2_. J. Am. Chem. Soc..

[40] Li J., Tang Z., Zhang Z. (2005). Preparation and novel lithium intercalation properties of titanium oxide nanotubes. Electrochem. Solide State Lett..

[41] Shin J.Y., Samuelis D., Maier J. (2011). Sustained lithium storage performance of hierarchical, nanoporous anatase TiO_2_ at high rates: Emphasis on interfacial storage phenomena. Adv. Funct. Mater..

[42] Li Y., Pan G.L., Liu J.W., Gao X.P. (2009). Preparation of Li_4_Ti_5_O_12_ nanorods as anode materials for lithium-ion batteries. J. Electrochem. Soc..

[43] Song H.J., Kim J.C., Roh H.S., Lee C.W., Park S., Kim D.W., Hong K.S. (2014). Anion controlled synthesis of TiO_2_ nano aggregates for Li Ion battery electrodes. Mat. Charac..

[44] Pan X., Yang M.Q., Fu X., Zhang N., Xu Y.J. (2013). Defect TiO_2_ with oxygen vacancies: Synthesis properties and photocatalytic applications. Nanoscale.

[45] Ventosa E., Madej E., Zampardi G., Mei B., Weide P., Antoni H., La Mantia F., Muhler M., Schuhmann W. (2017). Solid electrolyte interphase (SEI) at TiO_2_ electrodes in Li-ion batteries: Defining apparent and effective SEI based on evidence from X-ray photoemission spectroscopy and scanning electrochemical microscopy. ACS Appl. Mater. Interfaces.

[46] Lee K., Song S. (2011). One step hydrothermal synthesis of mesoporous anatase TiO_2_ microsphere and interfacial control for enhanced lithium storage performance. ACS Appl. Mater. Interfaces.

[47] Lin Z., Yue W., Huang D., Hu J., Zhang X., Yuan Z., Yang X. (2012). Pore length control of mesoporous Co_3_O_4_ and its influence on the capacity of porous electrodes for lithium-ion batteries. RSC Adv..

